# Millimeter-Wave Array Radar-Based Human Gait Recognition Using Multi-Channel Three-Dimensional Convolutional Neural Network

**DOI:** 10.3390/s20195466

**Published:** 2020-09-23

**Authors:** Xinrui Jiang, Ye Zhang, Qi Yang, Bin Deng, Hongqiang Wang

**Affiliations:** College of Electronic Science and Technology, National University of Defense Technology, Changsha 410073, China; Jane_nudt@163.com (X.J.); zhangye10@nudt.edu.cn (Y.Z.); dengbin@nudt.edu.cn (B.D.); wanghongqiang@nudt.edu.cn (H.W.)

**Keywords:** human gait recognition, millimeter-wave array radar, multi-channel three-dimensional convolution neural network, feature fusion

## Abstract

At present, there are two obvious problems in radar-based gait recognition. First, the traditional radar frequency band is difficult to meet the requirements of fine identification with due to its low carrier frequency and limited micro-Doppler resolution. Another significant problem is that radar signal processing is relatively complex, and the existing signal processing algorithms are poor in real-time usability, robustness and universality. This paper focuses on the two basic problems of human gait detection with radar and proposes a human gait classification and recognition method based on millimeter-wave array radar. Based on deep-learning technology, a multi-channel three-dimensional convolution neural network is proposed on the basis of improving the residual network, which completes the classification and recognition of human gait through the hierarchical extraction and fusion of multi-dimensional features. Taking the three-dimensional coordinates, motion speed and intensity of strong scattering points in the process of target motion as network inputs, multi-channel convolution is used to extract motion features, and the classification and recognition of typical daily actions are completed. The experimental results show that we have more than 92.5% recognition accuracy for common gait categories such as jogging and normal walking.

## 1. Introduction

Pedestrian retrieval and identification has always been an urgent need in the fields of anti-terrorism security checks, crime investigation, medical inference, etc. Due to the long distance between monitoring equipment and pedestrians, the commonly used biometric recognition methods such as face recognition and iris recognition cannot be applied to actual scenes. However, gait, a biological feature, can be effectively applied to pedestrian retrieval and recognition due to its characteristics of being unhidden, without disguise, non-invasive, stable, and remotely accessible [1,2].

Electromagnetic waves can provide long-distance detection of the human body and are not limited by time and climate conditions. Therefore, research on human body detection and feature extraction using radar has attracted more and more attention, which is a necessary supplement to traditional methods. Human gait information mainly refers to the micro-motion features of the human body, which contain effective information of the human motion state and body posture and are an important basis for human target authentication and recognition [3,4,5]. 

In 2018, Ann-Kathrin et al. studied motion classification based on radar micro-Doppler characteristics [6]. This paper presents a new classification method based on physical features, subspace features and harmonic models. By processing the echo data, analyzing the spectrogram (depicting Doppler and micro-Doppler signals corresponding to velocity and its time-varying properties) and the frequency–velocity gram (emphasizing periodicity and better describing the harmonic components of limbs), it is proved that different walking modes can be distinguished according to the results of feature extraction. In 2019, they used Doppler radar to monitor indoor human movements. The study found that by extracting Doppler signals, a slight difference in step size between the two legs can be detected, thus allowing the judgement of gait asymmetry and diagnosing whether the target human body has dyskinesia [7]. These related studies prove the feasibility of gait recognition using the micro-Doppler characteristics of radar signals.

In previous studies, radar-based gait recognition mainly relied on low-frequency radar with a single transmitting antenna and a single receiving antenna. A single antenna cannot carry out multi-dimensional detection and can provide less target feature information. At the same time, due to the low carrier frequency and small bandwidth, the resolution of radar is difficult to meet the requirements of high-precision identification with [8,9]. Meanwhile, traditional feature-extraction methods such as Jordan transform and principal component analysis (PCA) have poor real-time performance [10], and common classification algorithms such as invisible Markov model and support vector machine (SVM) are difficult to achieve high accuracy with [11,12,13]. Therefore, new feature-extraction and classification algorithms are urgently needed.

Millimeter-wave (MMW) radar works in the transition frequency band between microwaves and optics. It is easy to use to realize signals with large bandwidth and narrow beam, has an extremely high resolution, can obtain the fine structure and motion characteristics of targets, and has great application prospects in military and civil fields. MMW radar has two special advantages. The first advantage is high micro-Doppler sensitivity. That is, targets that cannot be distinguished by micro-Doppler differences in the traditional low-frequency band can be distinguished in the MMW band. Secondly, MMW radar can image targets with a high resolution and high frame rate, which makes it possible to retrieve and identify target characteristics with high precision based on images [14,15]. Combining MMW ultra-wideband signals with multiple-input multiple-output (MIMO) arrays can further obtain three-dimensional (3-D) spatial information on the detection area, thus obtaining a richer spatial distribution of targets [16].

The vigorous development of deep learning (DL) provides a unified framework for radar signal processing that integrates perception, processing and decision-making. Compared with the traditional pattern-recognition method, the DL method has the advantages of the automatic extraction of deep features and high recognition accuracy, and it has good universality. [17,18]. In recent years, the deep-learning (DL) technique has become a research hotspot in various fields, such as object classification and segmentation [19,20], super-resolution [21,22], image denoising [23,24], medical image reconstruction [25,26], etc. In addition to the above applications, it is also adopted in radar signal-processing applications. The typical cases include target detection [27], synthetic aperture radar(SAR)image interpretation [28] and moving-human-body classification [29,30].

Most gait recognition methods that combine radar sensors with DL mainly use convolution neural networks (CNN) to extract and recognize features of micro-Doppler signatures [6,7,31]. The birth of the 3-D spatiotemporal CNN provides a better method for processing the gait information of a time sequence [32,33,34]. It also provides more research directions for gait recognition technology. In 2018, Mingmin Zhao et al. of the Massachusetts Institute of Technology realized the through-wall identification of a target human body through radar sensors [35]. In this study, they used an optical collector and radar sensor to track and collect data of the target human body at the same time. The supervised learning framework based on the three-dimensional convolution neural network (3-D CNN) was used, and the obtained optical pictures and radar echo data were used for training. As a result, they realized the posture recognition of the human body in real time and effectively avoided the problem of recognition-rate decline caused by obstacle occlusion by using radar sensors. In 2019, this team proposed 3-D human pose estimation based on radar signals on the basis of previous research [36]. Their research results proved that the three-dimensional spatiotemporal convolution neural network had great advantages in processing spatiotemporal continuous gait data.

Based on the above analysis, this paper proposes a human gait recognition technology based on MMW array radar around the two basic problems of human gait detection by radar. After recording the echo data of array radar, we can use signal-processing methods such as linear filtering, time–frequency transformation, wavenumber domain compensation imaging and 3-D point cloud feature extraction to obtain abundant one-dimensional, two-dimensional and three-dimensional feature information, which can be used as input samples for the network. At the same time, a multi-channel three-dimensional convolution neural network (MC-3DCNN) is proposed based on improving the residual network (ResNet), which completes the classification and recognition of human gait through the hierarchical extraction and fusion of multi-dimensional features.

The rest of this paper is organized as follows. The generation of the point cloud of human gait is described in Section 2. Section 3 discusses the structure of the proposed MC-3DCNN. The training, analysis and comparison of the network are described in Section 4. The conclusions are drawn in Section 5. 

## 2. Generation of Point Cloud of Human Gait

### 2.1. Frequency-Modulated Continuous Wave and Range Measurement

Frequency-modulation continuous wave (FMCW) radar, which can measure the range, velocity and angle of the target by transmitting FMCWs, plays an important role in security and intelligent driving.

By mixing the received signal with the transmitted signal, we can obtain the intermediate frequency (IF) signal. Suppose the distance between the stationary target and the radar is R, the speed of electromagnetic wave is c and the slope of the chirp signal is K; then, the IF signal is expressed as:

(1) $$\begin{array}{cc} S_{IF} & =e^{i2\pi (K\frac{2R}{c}t)}\\ & =e^{i2\pi (f_{IF}t)} \end{array}$$

The above signal is a single-frequency signal (for a single stationary target). By Fourier transformation (FT), we can find out the position of peak and obtain the IF, $f_{IF}=K\frac{2R}{c}$; then, the distance of the target is expressed as:

(2) $$R=\frac{f_{IF}c}{2K}$$

For multiple targets, the IF signal is the superposition of multiple single-frequency signals. After the fast Fourier Transformation (FFT), there will be multiple peaks in the amplitude spectrum, and the frequency point of each peak is proportional to the distance between the target and the radar.

### 2.2. The Principle of Velocity Estimation

Supposing the target is located at $R_{0}$ in the initial time and is far away from the radar at a speed of v, the distance between the target and radar at time t is $R=R_{0}+vt$. The time delay τ can be expressed as $\tau =\frac{2R}{c}=\frac{2(R_{0}+vt)}{c}$, and the initial frequency of radar is $f_{0}$; then, the received signal $S_{r}$ can be expressed as:

(3) $$S_{r}=e^{j2\pi \left[f_{0}\left(t-\frac{2\left(R_{0}+vt\right)}{c}\right)+\frac{1}{2}K{\left(t-\frac{2\left(R_{0}+vt\right)}{c}\right)}^{2}\right]}$$

Due to the extremely short processing time (usually approximately ms or us), the term of $t^{2}$ can be ignored; at the same time, the term with $c^{2}$ in the denominator can also be ignored. Thus, the IF signal of the moving target can be expressed as

(4) $$S_{IF}=e^{j2\pi \left[\left(\frac{2R_{0}K+f_{0}v}{c}\right)t+\frac{2R_{0}f_{0}}{c}\right]}$$

Considering three practical situations under this model:

1. Time is discrete and determined by the sampling period $T_{s}$.
2. A total of N data are sampled per pulse.
3. L pulses are continuously emitted.
4. The radial component of the target velocity is constant v.

Modifying the mathematical model of the above IF signal, we can obtain:

(5) $$S_{IF}=e^{j2\pi \left[\left(\frac{2vf_{0}}{c}+\frac{2K\left(R_{0}+vlT_{c}\right)}{c}\right)nT_{s}+\frac{2\left(R_{0}+vlT_{c}\right)}{c}f_{0}\right]}$$

where n=0, 1, 2, …, N−1 means a single pulse sampling point sequence. $R_{0}$ denotes the radial distance between the radar and target at time 0 (the start time of the first radar pulse). l=0,1,2,…,L−1 represents the pulse sequence. $T_{c}$ denotes the pulse repetition time (the time difference between the start of two adjacent pulses).

Analysis of Equation (5) shows that for a specific pulse (l fixed), the $S_{IF}$ is still a single frequency signal. Compared with IF signal of a stationary target, it has a fixed value $e^{j2\pi \left[\left(\frac{2\left(R_{0}+vlT_{c}\right)}{c}f_{0}\right)\right]}$, which can be regarded as the complex envelope of the initial signal. The mathematical model of the initial signal can be expressed as:

(6) $$S_{in}=e^{j2\pi \left[\left(\frac{2vf_{0}}{c}+\frac{2K\left(R_{0}+vlT_{c}\right)}{c}\right)nT_{s}\right]}$$

Using n as the independent variable, the FFT of the signal in Equation (6) can be used to obtain the frequency component of the signal:

(7) $$f_{IF}=\frac{2vf_{0}}{c}+\frac{2K\left(R_{0}+vlT_{c}\right)}{c}$$

Taking l as the independent variable, the FFT for different pulses is equivalent to Fourier analysis for the phase components of the above signals, and the phase information of the signals can be obtained, which includes the speed of the target.

### 2.3. The Principle of DOA Estimation

**Figure 1:**
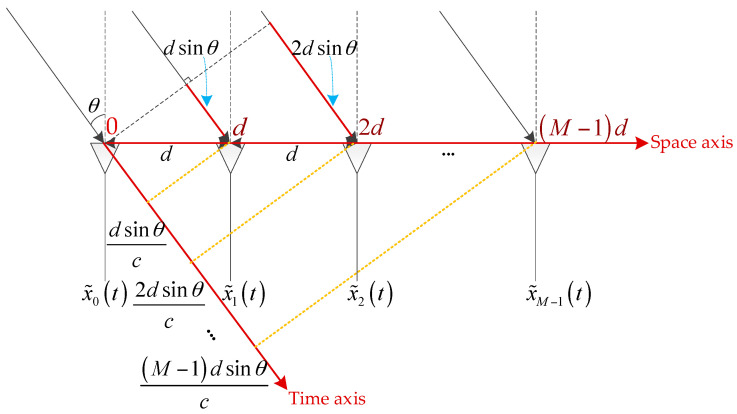
Geometric diagram of the relationship between the direction of arrival and array structure.

In the array radar system, we can estimate the direction of arrival (DOA) and obtain the spatial angle information of the target by using the spatial phase difference. As shown in the Figure 1, there is a uniform linear array with a total of M array elements, the distance between the array elements is d, and a signal (assumed to be a plane wave) is injected into the array from a direction θ away from the normal. It can be seen that the signal has to travel a further distance dsin(θ) to reach the second array element than to reach the first one, and so on. The signal has to travel a further distance dsin(θ) to reach the latter array element than the previous one.

After calculating the time difference of transmission, it can be concluded that for the same signal, the time to reach the latter array element is $\delta t=\frac{d\sin\left(\theta \right)}{c}$ later than that of the previous array element. Assuming that the frequency of the signal is $f_{0}$ and the first array element is taken as the reference point, the time difference between each array element and the first array element is:

(8) $$\Delta t=\left[0,\frac{d\sin\left(\theta \right)}{c},\frac{2d\sin\left(\theta \right)}{c},\cdots ,\frac{\left(M-1\right)d\sin\left(\theta \right)}{c}\right]$$

Then, the phase difference between the signals arriving at each array element and the first array element should be:

(9) $$\Delta \phi =\left[0,2\pi f_{0}\frac{d\sin\left(\theta \right)}{c},2\pi f_{0}\frac{2d\sin\left(\theta \right)}{c},\cdots ,2\pi f_{0}\frac{\left(M-1\right)d\sin\left(\theta \right)}{c}\right]$$

Since this phase difference is caused by different spatial positions between array elements, it is called the “spatial phase difference”.

In the time domain, the digital frequency can be extracted from the discrete time signal through FFT:

(10) $$y\left(n\right)=\left[\sin\left(2\pi f_{0}\times 0\right),\sin\left(2\pi f_{0}\times T_{s}\right),\sin\left(2\pi f_{0}\times 2T_{s}\right),\cdots ,\sin\left(2\pi f_{0}\times \left(N-1\right)T_{s}\right)\right]$$

and the extracted digital frequency is:

(11) $$\omega =2\pi f_{0}T_{s}=2\pi \frac{f_{0}}{f_{s}}$$

It is clearly seen from observation that this digital frequency is the phase difference $\Delta \phi =2\pi f_{0}T_{s}=2\pi \frac{f_{0}}{f_{s}}$ between adjacent sampling points.

Corresponding to the airspace, assuming that the far-field signal is s(n) and the first array element (numbered 0 in the above figure) of the array is taken as a reference, the signal received by the entire array is:

(12) $$X\left(n\right)=\left[s\left(n\right),s\left(n\right)e^{j2\pi f_{0}\frac{d\sin\left(\theta \right)}{c}},s\left(n\right)e^{j2\pi f_{0}\frac{2d\sin\left(\theta \right)}{c}},s\left(n\right)e^{j2\pi f_{0}\frac{3d\sin\left(\theta \right)}{c}},\cdots ,s\left(n\right)e^{j2\pi f_{0}\frac{\left(M-1\right)d\sin\left(\theta \right)}{c}}\right]$$

Through simple deformation, we can obtain:

(13) $$X\left(n\right)=\left[1,e^{j2\pi f_{0}\frac{d\sin\left(\theta \right)}{c}},e^{j2\pi f_{0}\frac{2d\sin\left(\theta \right)}{c}},e^{j2\pi f_{0}\frac{3d\sin\left(\theta \right)}{c}},\cdots ,e^{j2\pi f_{0}\frac{\left(M-1\right)d\sin\left(\theta \right)}{c}}\right]s\left(n\right)$$

It is clearly seen that the expression of received signal vector X(n) is a vector $\left[1,e^{j2\pi f_{0}\frac{d\sin\left(\theta \right)}{c}},e^{j2\pi f_{0}\frac{2d\sin\left(\theta \right)}{c}},e^{j2\pi f_{0}\frac{3d\sin\left(\theta \right)}{c}},\cdots ,e^{j2\pi f_{0}\frac{\left(M-1\right)d\sin\left(\theta \right)}{c}}\right]$ multiplied by a scalar s(n), and this vector is a function of the signal incoming direction θ. Define:

(14) $$a\left(\theta \right)≜\left[1,e^{j2\pi f_{0}\frac{d\sin\left(\theta \right)}{c}},e^{j2\pi f_{0}\frac{2d\sin\left(\theta \right)}{c}},e^{j2\pi f_{0}\frac{3d\sin\left(\theta \right)}{c}},\cdots ,e^{j2\pi f_{0}\frac{\left(M-1\right)d\sin\left(\theta \right)}{c}}\right]$$

The vector a(n) contains angle information of the signal s(n). The received signal can be represented as:

(15) X(n)=a(θ)s(n)

When the steering vector and the received signal are written as column vectors, the received signal can be expressed as:

(16) $$X{\left(n\right)}_{M\times 1}=a{\left(\theta \right)}_{M\times 1}s\left(n\right)$$

When N signals $s_{1}\left(n\right),s_{2}\left(n\right),\cdots ,s_{N}\left(n\right)$ are incident on the array from $\theta_{1},\theta_{2},\cdots ,\theta_{N}$ respectively, the received signal can be expressed as:

(17) $$\begin{array}{cc} X{\left(n\right)}_{M\times 1} & =a\left(\theta_{1}\right)s_{1}\left(n\right)+a\left(\theta_{2}\right)s_{2}\left(n\right)+\cdots +a\left(\theta_{N}\right)s_{N}\left(n\right)\\ & ={\left[a\left(\theta_{1}\right),a\left(\theta_{2}\right),\cdots ,a\left(\theta_{N}\right)\right]}_{M\times N}\times {\left[s_{1}\left(n\right),s_{2}\left(n\right),\cdots ,s_{N}\left(n\right)\right]}_{1\times N}^{T}\\ & ≜A_{M\times N}s_{N\times 1} \end{array}$$

Constructing a steering vector with an incoming direction of α:

(18) $$a\left(\alpha \right)≜\left[1,e^{-j2\pi f_{0}\frac{d\sin\left(\alpha \right)}{c}},e^{-j2\pi f_{0}\frac{2d\sin\left(\alpha \right)}{c}},e^{-j2\pi f_{0}\frac{3d\sin\left(\alpha \right)}{c}},\cdots ,e^{-j2\pi f_{0}\frac{\left(M-1\right)d\sin\left(\alpha \right)}{c}}\right]$$

The vector inner product of the steering vector a(α) and the received signal is obtained:

(19) $$y=a^{H}\left(\alpha \right)\cdot X\left(n\right)=a^{H}\left(\alpha \right)a\left(\theta \right)s\left(n\right)$$

The result is a scalar, and by calculating, we can obtain:

(20) $$y=\left[1+e^{j2\pi f_{0}d\frac{\sin\left(\alpha \right)-\sin\left(\theta \right)}{c}}+e^{j2\pi f_{0}d\frac{2\left[\sin\left(\alpha \right)-\sin\left(\theta \right)\right]}{c}}+\cdots +e^{j2\pi f_{0}d\frac{\left(M-1\right)\left[\sin\left(\alpha \right)-\sin\left(\theta \right)\right]}{c}}\right]s\left(n\right)\leq Ms\left(n\right)$$

The equal sign holds when α=θ.

Here, we introduce a DOA estimation method (see Table 1).

According to the above method, we can accurately estimate the direction of arrival and thus determine the direction of the target.

### 2.4. System and Generation of Point Cloud

**Figure 2:**
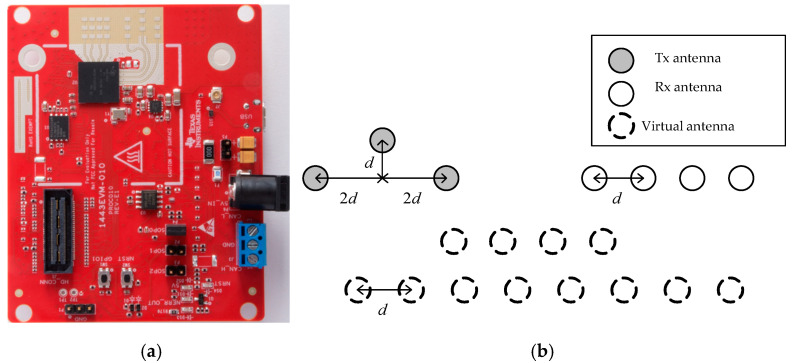
Radar system: (**a**) Structure of radar system; (**b**) Antenna array element and virtual aperture.

In this study, a 77 GHz FMCW array radar was used as the system platform, which is shown in (a) of Figure 2. The radar has three transmitting antennas and four receiving antennas, which can be equivalent to 12 virtual apertures according to the principle of equivalent phase centers, as shown in (b) of Figure 2.

The MMW array radar has an initial frequency of 77 GHz and a bandwidth of 4 GHz, which can provide better range resolution, while multiple aperture detection provides more azimuth angle data. At the same time, the radar has its own digital signal processor (DSP), which can carry out FFT on echo data in multiple dimensions, thus completing the ranging, velocity measurement and angle measurement of the target.

**Figure 3:**
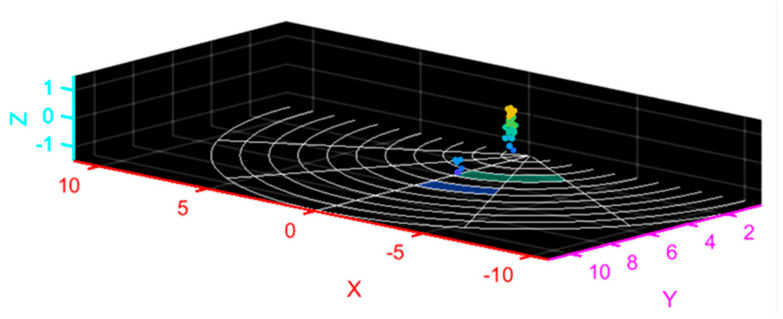
Point cloud collection.

In this study, radar was used to quickly track and locate the human body, and point cloud data of strong scattering points of the human body were obtained in real time through algorithm processing, which is shown in Figure 3. The point cloud data include the 3-D spatial coordinates, radial velocity and intensity of each scattering point.

Through parameter setting, the radar can collect 20 frames of point cloud data per second; each frame contains 0–64 point clouds of strong scattering points of the human body, and each point cloud is a five-dimensional (5-D) array composed of the 3-D spatial coordinates, radial velocity and intensity of the point.

Generally speaking, the human gait period is 1–1.32 s, and each gait period contains a series of typical posture shifts. In the process of walking, the trunk of each part of the human body moves regularly, and the movement characteristics of each strong scattering point of the human body also show regular changes. The acquired data maintain certain continuity in time and space, and the motion characteristics of the target can be grasped by calculating and analyzing the motion data. The 3-D spatial coordinates, radial velocity and intensity of strong scattering points in the motion process are obtained, and the feature extraction of the data sequence is carried out by using spatiotemporal convolution, thus completing the classification and recognition of human gait.

## 3. Human Gait Recognition Based on Multi-Channel 3-D Convolutional Neural Network 

The convolution neural network (CNN) has enabled great achievements in the field of image recognition. Using this model, image features can be extracted. In video classification, the traditional method is to extract the features of each key frame by using two-dimensional (2-D) CNN and then combine the features of the key frames by using relevant algorithms. There is a problem in this method: when using 2-D CNN, each frame of image is taken as a still picture for feature extraction, and the motion information in the time dimension is lost.

3-D CNN based on spatiotemporal convolution has been proved to be an effective spatiotemporal feature-extraction method. It extracts static information while retaining motion information and has achieved good results in video classification and other tasks.

In this study, we used the point cloud data of human gait as training samples. The point cloud data of human gait have certain continuity in their time and space distribution, which can be regarded as continuous frames in video classification. Therefore, the 3-D CNN can also be applied to human gait recognition.

### 3.1. Three-Dimensional Convolutional Neural Network

**Figure 4:**
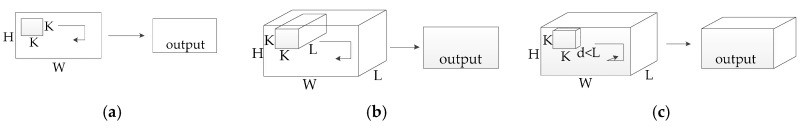
Diagram of convolution process [32]: (**a**) 2-D convolution; (**b**) 2-D convolution on multiple frames; (**c**) 3-D convolution.

Compared with the 2-D CNNs, the 3-D CNNs have better performance in time information modeling and spatiotemporal feature learning. In the 3-D CNNs, the convolution and pooling processes are completed in the space-time dimension, while in the 2-D CNNs, the convolution and pooling processes are only completed in space. Figure 4 shows the difference between the two network structures.

The three graphs in Figure 4 show the results of different convolution processing for different data. In (a), 2-D convolution is applied to an image (2-D array), and the output is an image (2-D array) too. In (b), 2-D convolution (multiple frames are regarded as multiple channels) is applied to a video (3-D array, including time series), and the output is an image (2-D array). In (c), the application of 3-D convolution on the video produces a 3-D array. Using 2-D convolution will cause the loss of the time information of the input signal after each operation, while 3-D convolution can retain the time information of the input signal.

Therefore, in this study, the method of 3-D convolution was used to extract spatiotemporal features, which include motion features in addition to static features and can achieve better results in gait classification tasks.

### 3.2. Proposed Network Architecture

**Figure 5:**
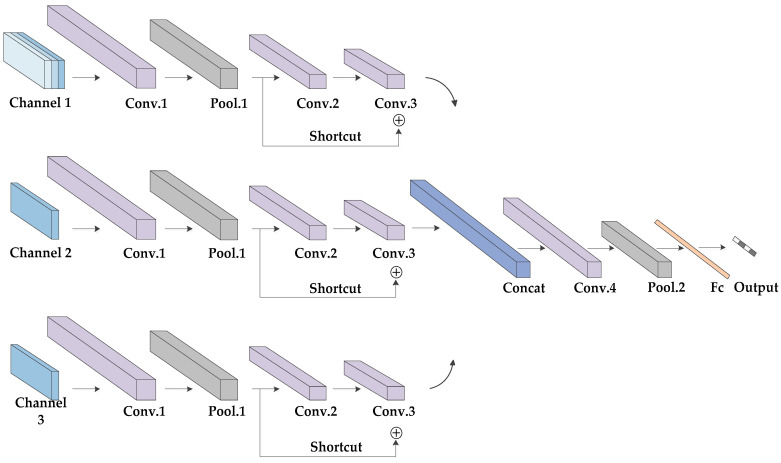
Structure of multi-channel three-dimensional convolution neural network (MC-3DCNN).

As shown in Figure 5, the proposed multi-channel three-dimensional convolution neural network (MC-3DCNN) consists of three channels, whose inputs are the 3-D spatial coordinates, radial velocity and intensity of the scattering points, respectively. In addition, each channel shares the same network topology, including three convolution layers and one pooling layer. The concatenation layer is used to fuse the features extracted hierarchically, and then, one convolution and one pooling layer are used to further extract and reduce the dimensions of the data and the fully-connected layer is then used to complete the classification. Finally, the classification results are given by the softmax classifier. The weights of each channel are updated independently to ensure that the characteristics of different sample data are fully learned.

Three channels are set up in the network; one channel takes 3-D spatial coordinates as input, and the other two channels take radial velocity and intensity as input, respectively. Considering the symmetry of the network structure, we will introduce it in detail with the structure of Channel 1 as the representative. First, we segment the gait sequence samples evenly. Each group of gait data contains multiple gait periods and can be divided into multiple gait segments. The gait period of the human body is usually 1–1.32 s. Here, we set the duration of each gait segment to be 2 s, including a complete gait period. Since the sampling rate is 20 frames per second, the gait data are taken as a training sample every 40 frames (2 s). Therefore, the input sizes are 3 × 64 × 40, 1 × 64 × 40 and 1 × 64×40, respectively. The kernel size of the first convolutional layer is set to 3 × 3 × 3, and the stride size, to 1 × 1 × 1, generating 64 feature maps. Then, these feature maps are subsampled by the max pooling layer with a kernel size of 3 × 3 × 3 and stride size of 2 × 2 × 2. The kernel size of the second convolutional layer is 3 × 3 × 3, and that of the stride is 2 × 2 × 1, generating 128 feature maps. The kernel size of the third convolutional layer is 3 × 3 × 3, and the stride size is 1 × 1 × 1. Here, in order to fit the function better and avoid the gradient’s disappearance, we use the structure of a residual network for reference and add a shortcut channel. The shortcut channel includes a convolution with a kernel size of 3 × 3 × 3 and stride size of 2 × 2 × 1. Then, the convolution results of each channel are fused through a concatenation layer to form new feature maps. The kernel size of the forth convolution layers is 3 × 3 × 3, and the stride size is 2 × 2 × 2, generating 64 feature maps. The following is an average pooling layer with a kernel size of 3 × 3 × 3 and stride size of 2 × 2 × 2. We use the flatten function to expand the obtained feature maps and use these as inputs of the fully connected layer. Here, in order to avoid the over-fitting of the network, we set up a dropout layer. In addition, a batch normalization (BN) layer was added after each convolutional layer, and all the activation function is ReLU.

For channel 1, channel 2 and channel 3, the output vectors of the third convolutional layers are defined as $F^{1}=\left(f_{1}^{1},f_{2}^{1},\cdots ,f_{N_{1}}^{1}\right)$, $F^{2}=\left(f_{1}^{2},f_{2}^{2},\cdots ,f_{N_{2}}^{2}\right)$ and $F^{3}=\left(f_{1}^{3},f_{2}^{3},\cdots ,f_{N_{3}}^{3}\right)$, respectively. Then the concatenation operation in concatenation layer is defined as:

(21) $$o=F^{1}⊕F^{2}⊕F^{3}≜\left(f_{1}^{1},f_{2}^{1},\cdots ,f_{N_{1}}^{1},f_{1}^{2},f_{2}^{2},\cdots ,f_{N_{2}}^{2},f_{1}^{3},f_{2}^{3},\cdots ,f_{N_{3}}^{3}\right)$$

where $N_{1}=N_{2}=N_{3}$ is the number of elements in the vector.

During the backpropagation, we define the cross-entropy loss function as:

(22) $$C=-\frac{1}{N}\sum_{i=1}^{N}\sum_{j=1}^{K}y_{ij}\log({\hat{y}}_{ij})$$

where N denotes the number of samples, K represents the number of categories, $y_{ij}$ is the expected probability that sample i belongs to category j, and ${\hat{y}}_{ij}$ denotes the actual output probability of sample i belongs to category j.

## 4. Experimental Results

### 4.1. Dataset Generation

**Figure 6:**
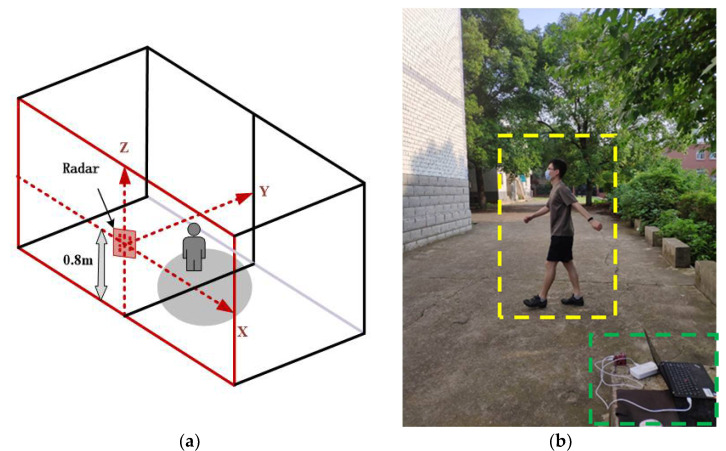
Experimental scenario: (**a**) Schematic diagram of experimental scene; (**b**) Real experimental scene.

In order to classify and recognize human gait accurately, we used a 3Tx-4Rx MMW array radar to detect targets and obtained gait data in various actual scenes, and the radar model was IWR1443BOOST from Texas Instruments. The experimental scene is shown in Figure 6. The radar was placed at a height of 0.8 m from the ground, and the target moved along an unfixed path within the radar detection range as shown in (a) of Figure 6. The radar detected the human body through scanning and used fast algorithms to realize range, speed and angle measurements of strong scattering points of the human body, thus realizing fast tracking and positioning. The obtained data were point cloud data including the 3-D spatial coordinates, radial velocity and intensity of strong scattering points.

The target moved along an unfixed path in the radar detection area, and the moving modes were normal walking (natural swing of both arms), jogging (raised forearm and natural swing of big arm), lame walking (normal walking for one leg, the dragging of one leg behind, and a slight swing of both arms) and squatting down and standing up. In order to increase the diversity of the sample data and enhance the robustness of the network model, in the experiment, we detected the human gait in various scenes (including corridors, basketball courts and parking lots). Each person collected 5000 frames of data for each type of action, and a total of eight different people (four men and four women) were observed. A total of 40,000 frames of sample data were collected for each type of action. The specific sample number and label division are shown in the Table 2.

**Figure 7:**
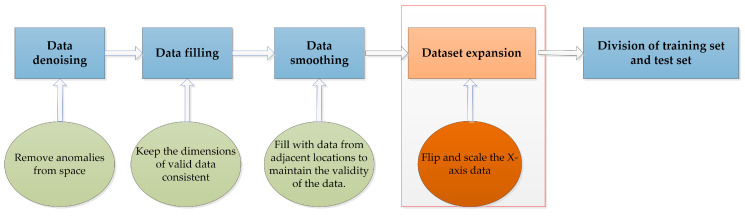
Flow of data processing.

The starting frequency of the radar is 77 GHz, and the bandwidth is 4 GHz. Through parameter setting, the radar can achieve a sampling rate of 20 frames per second, and each frame of data contains multi-dimensional data for 0–64 point clouds. The CNN realizes feature extraction by sliding convolution on the data matrix according to a certain step size through a convolution kernel. In this process, the input data are required to be a matrix with row and column rules. However, in this experiment, the data collection was random, that is, the number of point clouds in each frame of data was not always consistent. Therefore, it was necessary to preprocess the collected point cloud data and fill the data dimensions into regular shapes that were suitable for convolution network input. The main steps of preprocessing included data denoising, data filling, data smoothing, dataset expansion, and the division of the training set and test set. The specific operation flow is shown in Figure 7. The specific implementation method was as follows: firstly, a distance threshold was set in the 3-D space, and some noise points or outliers and other miscellaneous data in the data could be eliminated through the distance threshold, thus realizing the denoising of the data. Then, the dimensions of frames with few points were expanded to ensure the consistency of the input data dimensions. Here, we used null values to fill in, so that each frame of data contained 64 point clouds, thus completing the data filling. After dimension expansion, the point cloud data could not be null, because null values cannot provide useful data information for the convolution process. Therefore, we needed to copy the data of adjacent points, so that each frame of the point cloud was a 5-D array containing 64 valid points; thus, we realize data smoothing. In the case of small samples, it is necessary to expand the dataset by means of flipping and cropping, and then, the training set and the test set can be divided. The specific data preprocessing process is shown in the following figure.

In order to evaluate the network model, we adopted a new data partitioning strategy: N-fold crossover, which can avoid the limitations and particularity brought by the fixed partitioning of datasets. In the experiment, we divided the sample data into four folds, that is, as shown for each category, we divided the sample data into four equal parts for four-fold cross-validation (CV), as shown in Table 3. The division results for the training set and test set are shown in Table 4.

### 4.2. Classification Results and Algorithm Comparison

**Figure 8:**
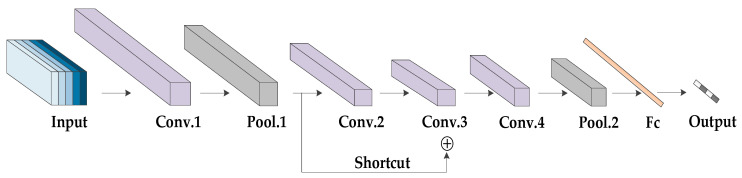
The structure of single-channel three-dimensional convolutional neural network (SC-3DCNN).

In the network training, we used a desktop computer with the Windows 10 system for training. The computer was equipped with a TITAN XP graphics card. For each cross-validation group, the training lasted for 60 epochs, and each training took approximately 2 h. The initial learning rate of the network is $1e^{-5}$, which degrades to 90% after every 20,000 iterations. To demonstrate that the proposed MC-3DCNN achieves higher recognition accuracy by enhancing and the fusion of features for 3-D spatial coordinates, radial velocity and intensity in the three channels, rather than increasing the number of training samples, we designed an experiment for a single-channel three-dimensional convolutional neural network (SC-3DCNN). The structure of the SC-3DCNN is shown in Figure 8, which has similar structural parameters to the MC-3DCNN. The SC-3DCNN integrates the 3-D spatial coordinates, radial velocity and intensity of every 40 frames of data into one sample as the input of the network. 

**Figure 9:**
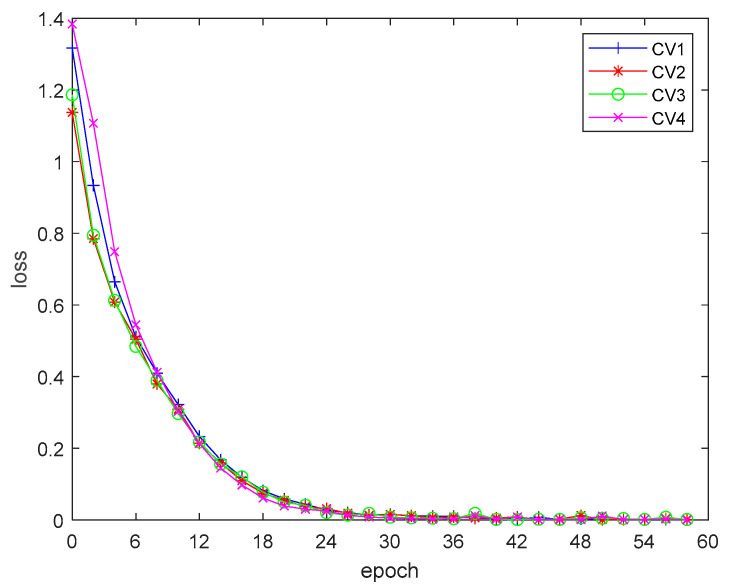
Training loss during cross-training.

**Figure 10:**
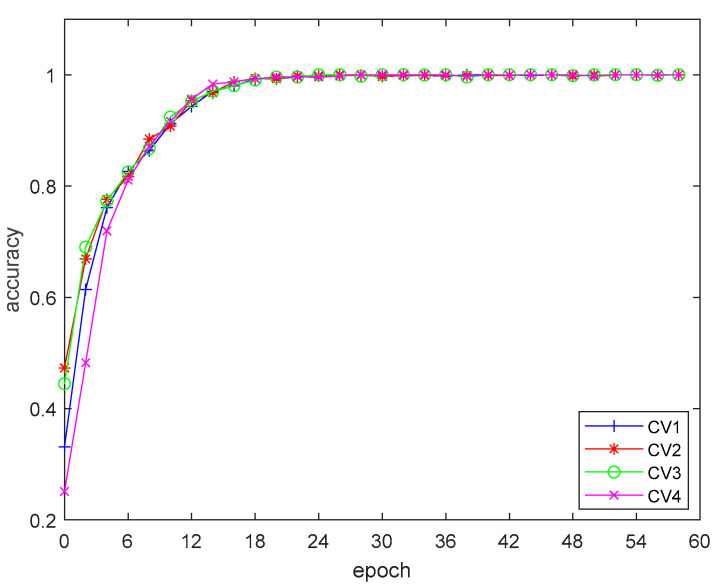
Training accuracy during cross-training.

The results of the four-fold cross-training are shown in Figure 9 and Figure 10. It can be seen from the training results that the network structure and parameter setting fit the distribution of the training data well, thus obtaining good training results in the four-fold cross training. The accuracy of the network continuously improved in the training process, and the accuracy of the network had reached more than 95% after 30 epochs of training. The training error converged smoothly, and after 30 epochs of training, the error converged to close to 0.

The trained network models were used for four-fold cross-validation to test the recognition accuracies of the network model for various actions. The recognition results are shown in the data in Table 5.

From the data in the table, it can be seen that the MC-3DCNN can better identify the three types of movements with strong continuity of movements and obvious micro-Doppler characteristics, including jogging, normal walking, squatting, and standing up, with an average accuracy rate of more than 92%. However, for the gait category with weak movement continuity and less obvious micro-Doppler characteristics, such as lame walking, the recognition accuracy is lower than that for the other three movements.

The four-fold cross-validation was carried out on the SC-3DCNN and MC-3DCNN, and the average verification results of each cross-validation are shown in the following Table 6.

The recognition results demonstrate that the proposed MC-3DCNN outperforms SC-3DCNN and achieves higher recognition accuracy by extracting features independently of multiple channels. 

## 5. Conclusions

In this paper, an MMW array radar-based human gait recognition method using MC-3DCNN is proposed. In this work, we used a 77 GHz MMW array radar as the sensor and used a multi-dimensional array antenna to obtain multi-dimensional information on the target. At the same time, in order to fuse multi-dimensional features, we propose a MC-3DCNN based on the 3-D CNN. The 3-D spatial coordinates, radial velocity and intensity of the observed human gait were extracted with hierarchical features, and then, the classification and recognition of human gait were completed by using multi-dimensional feature fusion. The network model proposed by us can achieve more than 92.5% recognition accuracy for daily travel modes such as jogging and normal walking.

Future work can be carried out on the following aspects: First, the recognition at this stage is mainly to classify the gait of the human. The next step could complete the identification of the human under the premise of a large-scale expansion of the sample database. Second, the current target of gait recognition is mainly a single human, and the next step would be mainly gait segmentation and classification recognition in complex scenes with multiple targets. Third, the current design is mainly for simple target gait category classification and identification; the next step could be carried out in the direction of using gait changes to analyze the intention of the perpetrator, which would play a positive role in gait recognition in the field of security inspection.

## Figures and Tables

**Table 1:** Direction of arrival (DOA) estimation method.

For: θ $\;\mathrm{from}-90^{o}$ $\;\mathrm{to}\;90^{o}$
calculate $y_{\alpha }=a^{H}\left(\alpha \right)\cdot X\left(n\right)$;
end for $\theta =\underset{\alpha }{\max}y_{\alpha }$

**Table 2:** Samples and labels.

Category of Action	Label of Action	Number of Samples (Frame)
normal walking	0	40,000
jogging	1	40,000
lame walking	2	40,000
squatting down and standing up	3	40,000

**Table 3:** Capacity of the dataset.

Dataset	Number (Frame)
Dataset 1	10,000 × 4
Dataset 2	10,000 × 4
Dataset 3	10,000 × 4
Dataset 4	10,000 × 4

**Table 4:** Cross-training and dataset partition.

Cross-Validation	Dataset 1	Dataset 2	Dataset 3	Dataset 4
CV_1	Training	Training	Training	Testing
CV_2	Training	Training	Testing	Training
CV_3	Training	Testing	Training	Training
CV_4	Testing	Training	Training	Training

**Table 5:** Recognition results of cross validation.

	Accuracy (%)	CV_1	CV_2	CV_3	CV_4	Average Accuracy
Category						
Jogging		95.20	89.80	94.60	90.40	92.50
Normal walking		90.40	95.20	96.80	89.60	93.00
Lame walking		81.60	94.20	89.60	85.60	87.75
Squatting down and standing up		94.80	92.50	89.80	93.60	92.68

**Table 6:** Recognition results for different networks.

	Accuracy (%)	CV_1	CV_2	CV_3	CV_4	Average Accuracy
Network						
SC-3DCNN		84.20	89.80	81.60	89.60	86.30
MC-3DCNN		90.50	92.93	92.70	89.80	93.00
